# Application of drug-induced sleep endoscopy in predicting the outcomes of velopharyngeal surgery in adult patients with Friedman stage II and III obstructive sleep apnea syndrome

**DOI:** 10.3389/fneur.2022.1049425

**Published:** 2023-01-09

**Authors:** Xin Cao, Yingqian Zhou, Junbo Zhang, Guoping Yin, Jingying Ye

**Affiliations:** ^1^Department of Otolaryngology, Head and Neck Surgery, Beijing Tsinghua Changgung Hospital, School of Clinical Medicine, Tsinghua University, Beijing, China; ^2^Department of Otolaryngology, Head and Neck Surgery, Peking University First Hospital, Beijing, China

**Keywords:** drug-induced sleep endoscopy (DISE), obstructive sleep apnea, velopharyngeal surgery, Friedman stage, collapse pattern

## Abstract

**Objective:**

This study aimed to evaluate the predictive value of drug-induced sleep endoscopy (DISE) for the outcomes of velopharyngeal surgery in adult patients with Friedman stage II and III obstructive sleep apnea syndrome (OSAS).

**Methods:**

A total of 39 male OSAS patients with Friedman stage II and III were retrospectively analyzed. Subjects with an apnea-hypopnea index (AHI) > 5 events/h indicated by polysomnography (PSG) and typical symptoms, such as snoring, sleep apnea, and daytime sleepiness, were included in this study. All these patients underwent pre-operative DISE examinations and were treated by velopharyngeal surgery and evaluated by velum, oropharynx, tongue base, and epiglottis (VOTE) scoring system. Clinical, polysomnographic parameters (e.g., hypopnea, apnea, AHI, lowest oxygen saturation, etc.), cephalometric variables, and DISE findings were evaluated. The treatment outcomes were assessed by polysomnography at least 6 months after surgery.

**Results:**

All 39 patients showed complete velopharyngeal airway collapses during pre-operative DISE examinations. After surgery, the AHI was significantly improved from 50.2 ± 21.6 to 19.8 ± 19 events/h (*P* < 0.05). There were 23 responders (59.0%) and 16 non-responders (41.0%). The glossopharyngeal airway collapse degree (GA-CD) was significantly different between responders and non-responders (*P* < 0.05). The velopharyngeal airway collapse pattern (VA-CP) and GA-CD were independently predictive of treatment outcomes (both *P* < 0.05). Patients with non-lateral VA-CP and grade II GA-CD (collapse degree > 50%) had a significantly lower surgical success rate than those without (*P* < 0.05).

**Conclusion:**

The VA-CP and GA-CD in DISE examination are valuable for predicting the treatment outcomes of velopharyngeal surgery in patients with Friedman stage II and III OSAS. Patients with lateral VA-CP and grade I GA-CD are appropriate candidates for velopharyngeal surgery.

## Introduction

Obstructive sleep apnea syndrome (OSAS) is a common disease characterized by episodic upper airway collapse or narrowing during sleep, and it is associated with potential physical and psychological sequelae if untreated (1, 2). Continuous positive airway pressure (CPAP) is the first line of therapy for OSAS, while the adherence rate of CPAP is problematic (3–5). Velopharyngeal surgeries, such as uvulopalatopharyngoplasty (UPPP) (6) and transpalatal advancement pharyngoplasty (TAP) (7), may be used as alternative options due to the intolerability and poor compliance of CPAP. However, the outcomes of velopharyngeal surgeries are unfavorable. Previous studies suggested that the success rate of velopharyngeal surgeries can be improved by selecting proper candidates for the surgery. Various parameters and methods, including patient age, pre-operative apnea-hypopnea index (AHI), body mass index (BMI), Friedman staging based on tonsil size and palate position (8), and the sites of collapse or narrowing using drug-induced sleep endoscopy (9) (DISE), were employed to predict the outcomes of velopharyngeal surgeries. Among these parameters, Friedman staging (10) is a simple and feasible parameter. A meta-analysis suggested that Friedman stage I is a strong predictor of post-UPPP success (8). The surgical outcomes of patients with Friedman stage II and III OSAS were 40 and 8%, respectively. The DISE is used to select appropriate patients with Friedman stage II and III OSAS for improving outcomes of velopharyngeal surgery. The DISE is a dynamic evaluation method and may identify the site, pattern, and severity of airway collapse, which may affect the success rate of surgery. In this study, it was hypothesized that DISE may help to select appropriate patients and improve the post-operative outcomes of velopharyngeal surgery.

This study retrospectively evaluated patients with Friedman stage II and III OSAS who underwent pre-operative DISE and revised UPPP with uvula preservation (H-UPPP) examinations with or without TAP.

## Materials and methods

### Patients' selection

Between December 2018 and December 2020, 39 OSAS patients who were treated by velopharyngeal surgery in Beijing Tsinghua Changgung Hospital (Beijing, China) were retrospectively studied. These patients were diagnosed with OSAS based on polysomnography (PSG; Polysomnography Device CE, EMBLA N7000, Medcare, Iceland) (AHI >5 events/h) and typical symptoms (e.g., snoring, sleep apnea, and daytime sleepiness). The inclusion criteria were as follows: (1) age >18 years; (2) Friedman stage II or III; (3) CPAP refusal or intolerance; and (4) performing PSG examinations at least 6 months after surgery. “Six months after surgery” is internationally considered for assessment of post-operative efficacy. In addition, a period of 6 months is enough for wound scar healing and complete regeneration of the peripheral nerves. Moreover, other treatment modalities may be considered if the post-operative outcomes are poor. Patients who underwent CPAP therapy, those who had complete glossopharyngeal collapse during DISE examination, or those who had serious comorbid lung, neurological, cardiovascular, or psychiatric disorders were excluded. All velopharyngeal surgeries, including H-UPPP alone or in combination with TAP, were performed by an experienced surgeon. The surgical procedures have been previously published. This study was approved by the Human Research Ethics Committee of Beijing Tsinghua Changgung Hospital. The objective of the study was explained to the subjects, and their written informed consent was obtained.

### PSG examinations

The pre- and post-operative overnight PSG examinations were undertaken at the Sleep Medical Center of Beijing Tsinghua Changgung Hospital. The findings were analyzed by experienced technicians according to the latest scoring guidelines of the American Academy of Sleep Medicine published in 2012 (11). An apnea was scored when there was a complete cessation of airflow or a ≥ 90% reduction in the peak thermal sensor signal for at least 10 s. Hypopnea was scored when there was a ≥ 50% reduction in the nasal pressure signal for at least 10 s with oxygen desaturation of ≥ 3% or arousal. The AHI was calculated as the sum of the number of apneas and hypopneas per hour of sleep. The lowest oxygen saturation (LSAT) was also calculated for further analysis.

### Drug-induced sleep endoscopy

The drug-induced sleep endoscopy (American Amberland Model: EMBLA N7000) procedures were performed as previously described (9). Subjects were placed in the supine position on the operating bed, with the lights dimmed under the monitoring of standard PSG. All patients were induced to sleep by target-controlled infusion (TCI) of dexmedetomidine using a bolus of 1 mg/kg/h at the beginning of the examination, and then, the infusion rate was changed to 0.8 μg/kg/h to maintain sleep. Topical nasal decongestants and topical anesthetic (1:10,000 tetracaine) were applied to both nasal cavities. When stable stage 2 sleep (three continuous epochs of EEG scored N2) was achieved, an endoscope was inserted through the nose, and the upper airway was assessed by videos and dynamically monitored for more than 15 min after stable sleep.

The drug-induced sleep endoscopy videos were stored for analysis. In accordance with the recent European publication, the velum, oropharynx, tongue base, and epiglottis (VOTE) scoring system was utilized to determine velopharyngeal airway collapse patterns (VA-CPs), including lateral, anteroposterior, and concentric collapse. The glossopharyngeal airway collapse degree (GA-CD) was also assessed and classified as grade I (slight airway obstruction, 0–50%) and grade II (severe airway obstruction, 50% or more). This method simplifies the VOTE scale, which is easy to assess with a low error rate. To avoid errors by subjective factors, the blinded analysis was performed, and the same investigator evaluated the findings two times at different time points. If the findings were inconsistent, another experienced physician would re-evaluate the results and discuss them until they reach a consensus.

### Statistical analysis

The statistical analysis was performed using SPSS 17.0 software (IBM Corp., Armonk, NY, USA). Continuous data were presented as mean ± standard deviation. The unpaired Student's *t*-test was used to compare quantitative variables between different groups. Pearson's Chi-square test was used to compare categorical variables between different groups. The multivariate logistic regression analysis was employed to evaluate the significance of independent variables. There were basic indicators of linear regression equations, including both quantitative and qualitative indicators. *P* < 0.05 was considered statistically significant.

## Results

All 39 patients were men, with a mean age of 38.2 ± 8.6 (range, 23–60) years. All these patients showed complete velopharyngeal airway collapse during pre-operative DISE examinations. Their AHI decreased significantly from 50.2 ± 21.6 events/h pre-operatively to 19.8 ± 19 events/h post-operatively (*P* < 0.05). According to the classical definition, subjects with ≥ 50% reduction in AHI to a final AHI of < 20 corresponded to successful surgical treatment, and patients were classified as responders. In contrast, subjects with < 50% reduction in AHI were regarded as non-responders (12, 13). In the present study, 23 (59.0%) patients achieved treatment success as defined by a ≥ 50% reduction in AHI to a final AHI of <20 and were regarded as responders; the remaining 16 (41.0%) patients were classified as non-responders. The pre-operative data and surgical outcomes of 39 patients are shown in Table 1, demonstrating a significant difference in GA-CD between the responder group and the non-responder group (*P* = 0.018). Other factors, including age, BMI, AHI, LSAT, surgical procedures, and VA-CP, showed no significant differences between the two groups (all *P* > 0.05).

**Table 1:** The pre-operative and surgical data of 39 patients according to treatment outcomes.

	**Responders, *n* = 23**	**Non-responders, *n* = 16**	***T* or x*^2^* value**	***P*-value**
Age (years old)	38.9 ± 9.5	37.3 ± 7.5	−0.573	0.57
BMI (kg/m^2^)	27.1 ± 3.0	27.4 ± 4.5	0.236	0.816
AHI (events/h)	49.0 ± 18.5	51.9 ± 25.8	0.385	0.704
LSAT (%)	76.3 ± 10.5	74.9 ± 12.7	−0.359	0.722
**Surgical procedures**				
H-UPPP	15	11	0.053	0.818
H-UPPP+TAP	8	5		
**VA-CP**				
Lateral	8	1	5.662	0.059
Anteroposterior	11	8		
Concentric	4	7		
**GA-CD**				
Grade I	13	3	5.564	0.018^*^
Grade II	10	13		

BMI, body mass index; AHI, apnea-hypopnea index; LSAT, lowest oxygen saturation; H-UPPP, revised uvulopalatopharyngoplasty with uvula preservation; TAP, transpalatal advancement pharyngoplasty; VA-CP, velopharyngeal airway collapse pattern; GA-CD, glossopharyngeal airway collapse degree.

* Statistically significant.

The multivariate logistic regression analysis that included all factors in Table 1 was performed to predict the success rate of surgery. The results suggested that VA-CP (*P* = 0.018) and GA-CD (*P* = 0.017) both independently predicted the success of the surgery. Their odds ratio (OR) values were 3.127 and −2.118, with 95% confidence intervals (CI) of 1.668–311.622 and 0.023–0.637, respectively.

The predictive values of the combination of VA-CP and GA-CD were further evaluated for treatment outcomes. As shown in Table 2, the results suggested that the success rate of patients with non-lateral VA-CP and grade II GA-CD was only 23.5%, which was significantly lower than that of the other three groups (all *P* < 0.05).

**Table 2:** The efficacy of surgery of 39 patients classified according to VA-CP and GA-CD.

	**VA-CP**		**Total**
	**Lateral**	**Non-lateral**	
**GA-CD**			
Grade I	66.7% (2/3)	84.6% (11/13)	81.3% (13/16)
Grade II	100% (6/6)	23.5% (4/17)	43.5% (10/23)
Total	88.9% (8/9)	50% (15/30)	59% (23/39)

## Discussion

The application of DISE in male patients with Friedman stage II and III OSAS before velopharyngoplasty procedures, such as H-UPPP with or without TAP, significantly improved the AHI of patients, even in those patients with multilevel obstruction. In this study, all subjects had complete velopharyngeal obstruction (100%), and VA-CP revealed a complete absence of constant tongue obstruction. It was observed that the hypopharyngeal soft tissues were more likely to collapse with respiratory movement after velopharyngeal airway blockage. Thus, the GA-CD was assessed in this study regardless of the collapse pattern of the hypopharyngeal airway. The GA-CD may indirectly reflect the potential of upper airway collapse. The different levels of upper airway collapse may result from factors, such as arousal threshold, obstructive length, and neural drive. The above-mentioned method may simplify the VOTE scale.

### Collapse pattern of the velopharyngeal airway

It was found that the VA-CP may be associated with surgical outcomes. The lateral collapse predicts the success rate of surgery, which explains why the H-UPPP or TAP may treat obstruction related to the lateral wall. AP collapse and concentric collapse predicted poor surgical outcomes in this study. Similar findings were also reported in other studies. Koutsourelakis et al. (14) described that circumferential collapse at the velar level and complete anteroposterior collapse of tongue base or epiglottis were independent predictors of failure of upper airway surgery. Ying-Shuo et al. (15) demonstrated that circumferential collapse at the velar level was associated with a higher residual AHI and a reduced AHI response to palatopharyngoplasty.

### Collapse degree of hypopharyngeal airway

The majority of OSAS patients show hypopharyngeal airway narrowing during DISE examinations. In the present study, 92.3% (36/39) of patients showed varying degrees of hypopharyngeal airway collapse without complete obstruction (100% collapse, which persisted for several seconds). Soares et al. (16) reported a higher possibility of retrolingual obstruction during DISE than during Müller's maneuver in patients with Friedman stage II and III OSAS. Gideon Bachar et al. found that laryngeal obstruction was typically supraglottic, and hypopharyngeal obstruction involved concentric upper airway narrowing, which could explain the high risk of surgical failure (17). Previous studies showed that the prevalence of complete, multilevel, and hypopharyngeal collapse increased with the severity of OSAS. This phenomenon may be associated with the pathogenic mechanism of OSAS. Tensor palatini and geniohyoid are likely hypotonic during sleep, which might result in pharyngeal closure in patients with OSAS (18). A narrowed segment of the passive airway is closed by force generated by relevant dilator muscles during sleep. Velopharyngeal surgeries may enlarge the airway by resectioning the narrowed segments, especially narrowed velopharyngeal airway tissues.

Hypopharyngeal airway obstruction serves as an important predictor of outcomes of upper airway surgery (19). The collapse of the hypopharyngeal airway is unlikely to completely obstruct the airway for several seconds and may lead to obstructive apnea events, while the partial obstruction or secondary collapse caused by respiratory movement is more common in patients with OSAS, which indirectly reflects the collapsibility and the obstructive strength of the upper airway.

A retrospective, multi-center cohort study (20) of adults without tonsillar hypertrophy who underwent pharyngeal surgery for OSAS demonstrated that the obstruction related to the oropharyngeal lateral wall was associated with poorer surgical outcomes. In contrast, complete tongue-related obstruction was correlated with a lower likelihood of surgical responses in moderate-to-severe patients with OSAS. In the present study, the obstruction related to the oropharyngeal lateral wall and tongue was included in the hypopharyngeal airway collapse, and the degree of collapse affected the surgical outcomes. The hypopharyngeal obstruction in the failure group was more severe. Thus, it may play an important role in predicting surgical outcomes by identifying the severity of hypopharyngeal obstruction, which was rarely reported in previous studies and hence should receive further attention in future studies.

There were some limitations to this study. First, the VOTE evaluation during DISE was subjective. Nevertheless, good consistency was confirmed for the results of VOTE. Second, the DISE-associated parameters were simplified and errors were minimized. Third, the small sample size is noteworthy. The sample investigated in the current research is an approximate estimate of the total size. Further investigation is still in progress, and randomized clinical trials on larger populations and updated research indicators may be included in the follow-up studies.

## Conclusion

The velopharyngeal airway collapse pattern and GA-CD in DISE examination were found as independent predictors of treatment outcomes of velopharyngeal surgery in patients with Friedman stage II and III OSAS. The lateral collapse of the velopharyngeal airway, regardless of the hypopharyngeal collapse, and slight hypopharyngeal collapse in patients with anteroposterior collapse or concentric collapse of the velopharyngeal airway may predict a better surgical outcome.

## Data availability statement

The raw data supporting the conclusions of this article will be made available by the authors, without undue reservation.

## Ethics statement

The studies involving human participants were reviewed and approved by the Ethics Committee of Beijing Tsinghua Changgung Hospital. Written informed consent for participation was not required for this study in accordance with the national legislation and the institutional requirements.

## Author contributions

GY and JY designed the study and revised the manuscript. XC and JZ participated in the material preparation, data collection, data analysis, and drafting of the manuscript. All authors commented on previous versions of the manuscript, read, and approved the final manuscript.
